# Cardiac miR-133a overexpression prevents early cardiac fibrosis in diabetes

**DOI:** 10.1111/jcmm.12218

**Published:** 2014-01-16

**Authors:** Shali Chen, Prasanth Puthanveetil, Biao Feng, Scot J Matkovich, Gerald W Dorn II, Subrata Chakrabarti

**Affiliations:** a Department of Pathology, Schulich School of Medicine and Dentistry, Western UniversityLondon, ON, Canada; b Department of Medicine, Center for Pharmacogenomics, Washington University School of MedicineSt. Louis, MO, USA

**Keywords:** diabetes, cardiac fibrosis, miRNA 133

## Abstract

Diabetic cardiomyopathy is a cascade of complex events leading to eventual failure of the heart and cardiac fibrosis being considered as one of its major causes. miR-133a is one of the most abundantly expressed microRNAs in the heart. We investigated the role of miR-133a during severe hyperglycaemia. And, our aim was to find out what role miR-133a plays during diabetes-induced cardiac fibrosis. We saw a drastic decrease in miR-133a expression in the hearts of streptozotocin-induced diabetic animals, as measured by RT-qPCR. This decrease was accompanied by an increase in the transcriptional co-activator EP300 mRNA and major markers of fibrosis [transforming growth factor-β1, connective tissue growth factor, fibronectin (FN1) and COL4A1]; in addition, focal cardiac fibrosis assessed by Masson's trichome stain was increased. Interestingly, in diabetic mice with cardiac-specific miR-133aa overexpression, cardiac fibrosis was significantly decreased, as observed by RT-qPCR and immunoblotting of COL4A1, ELISA for FN1 and microscopic examination. Furthermore, Cardiac miR-133a overexpression prevented ERK1/2 and SMAD-2 phosphorylation. These findings show that miR-133a could be a potential therapeutic target for diabetes-induced cardiac fibrosis and related cardiac dysfunction.

## Introduction

Diabetic cardiomyopathy is a complex condition mediated by tissue-specific and diabetes-related interconnected pathological processes. Focal cardiac fibrosis, a prominent cause for diabetic cardiomyopathy, is considered to be an early event, which sets the stage for heart failure by reducing contractile efficiency and demanding greater cardiac contractile force, eventually leading to cellular death [1, 2]. The key determinants of tissue fibrosis are an activated transforming growth factor-β (TGF-β) signalling cascade and accumulation of increased extracellular matrix (ECM) proteins such as fibronectin (FN1) and collagen 1 alpha 1V (COL4A1). The increased matrix protein accumulation could be due to a synergistic effect of TGF-β receptor stimulation, endothelin-1 (EDN1)-mediated downstream activation and the angiotensin (AT) pathway, all three of which have been shown to activate collagen production [3]. The ECM protein accumulation in diabetes results in an irreversible tissue damage [2], probably because of a result of tissue repair following cell death.

MicroRNAs (miRs) are small non-coding RNAs involved in virtually all biological processes, including angiogenesis, metabolism, cell growth, survival and death, proliferation, differentiation and pluripotency. Two miRs, which are selectively expressed in heart and which have been used as early markers of cardiac tissue damage, are miR-1 and miR-133a [3]. These miRs have been shown to have protective effects in cardiac tissue with response to hypertrophic stimuli. In particular, miRNA-133a has been shown to protect against myocardial fibrosis without affecting hypertrophy in pressure-overloaded adult hearts [4].

Given the significance of miR-133a as one of the predominantly expressed miRs in the cardiac tissue, and its prominent role in various events, starting from cardiac cell development to regulation of cell death and hypertrophy [4–5], makes miR-133a a therapeutic target in the heart, in diabetes. Furthermore, there are no reports exploring the role of miR-133a in fibrosis in response to chronic metabolic stress like diabetes. In this study, we have made use of the previously documented miR-133a transgenic (Tg) mice [4] to study the role of this miR in response to glucotoxic effects of diabetes, and explored the protective role of miR-133a in preventing cardiac fibrosis.

## Research design and methods

### Animals

All animals were housed and experiments conducted in accordance with the Declaration of Helsinki and the Guiding Principle in care and use of animals. All animal experimental protocols were approved by the University of Western Ontario Council on Animal Care Committee. Male mice were used for studies and induction of diabetes by streptozotocin was same as described previously [7–8].

### Treatments and transgenic mice strain

Streptozotocin was administered as 65 mg/kg i.p for three consecutive days, and glucose measurement and hyperglycaemic confirmation was performed and animals were kept for 2 months. The friend leukaemia virus B mice strain was used to study the role of miR-133a in the heart, by cloning the genomic sequence of miR-133a into the α-Myosin heavy-chain promoter that is cardiac specific as previously described [5].

### cDNA and protein isolation

At the end of 2 months, animals were anaesthetized and killed to isolate the hearts. Immunoblotting and qPCR studies were conducted as previously described [8, 10], with any modifications noted in figure legends. In brief, for cDNA, Trizol reagent was used for this extraction procedure (Invitrogen, Burlington, ON, Canada) using chloroform layer separation followed by treatment with isopropanol and ethanol. The cDNA from this RNA samples were produced using reverse transcriptase in a superscript II system (Invitrogen) using a polymerase chain reaction and stored at −20°C. For proteins, heart tissues were used and protein extracted using a buffer containing Tris (pH 7.5), 50 mmol/l NaCl, 1 mmol/l MgCl_2_, 5% glycerol, 0.05%, Nonidet P-40, 0.5 mmol/l ethylene diamine tetra acetic acid (EDTA), and 0.5 mmol/l dithiothreitol (DTT), and 0.5% *****Sodium Azide. The sample with buffer was sonicated and centrifuged at 14489 × ***g*** to isolate out the proteins and proteins were determined using Bradford protein assay method.

### Quantitative real-time PCR

The cDNA isolated was using for real-time PCR as described previously [8]. A well-established nucleic acid stain-*N′*,*N′*-dimethyl-*N*-[4-[(E)-(3-methyl-1,3-benzothiazol-2-ylidene)methyl]-1-phenylquinolin-1-ium-2-yl]-*N*-propylpropane-1,3-diamine (SYBR) dye method using a Roche Light Cycler was used to measure the required increase in target genes. In brief, 1 μl of sample cDNA, along with 10 μl of SYBR dye, 1.6 μl of MgCl_2_ and 5 μl of H_2_O was used in the reagent mixture. All the obtained CT values were normalized to 18S rRNA as the control or housekeeping gene.

### Western blotting and ELISA

After extracting proteins and performing the protein assay as described by a previously done method [8], 20 μg of proteins for each samples was subjected to SDS gel electropheresis, followed by Western blotting using primary antibodies of COL4A1, Phospho-SMAD, Total-SMAD, phosphor-extracellular signal–regulated kinase (ERK) and Total ERK. β-actin was used as a loading control. For estimation of Fibronectin, we used an ELISA kit (Abcam Inc, Toronto, ON, Canada) to measure the Fibronectin levels, where the sample is measured as an intensity of a horse radish peroxidase reaction given out by the yellow colour.

### Immunohistochemistry

The cardiac tissues were isolated from required groups and were sectioned at a thickness of 5 μM, and trichome staining was performed to measure the tissue fibrosis as described by a previously described method [11].

### Statistical analysis

Statistical difference was performed with one-way anova followed by a Tukey's method of comparison between groups, with a *P* < 0.05, and results are expressed as average of *n* = 6–8 animals per group ± SEM.

### Chemicals

All chemicals and reagents were obtained from Sigma-Chemicals (St. Louis, MO, USA) and antibodies from Santa Cruz Biotech Inc (Santa Cruz, CA, USA) and Cell Signaling Technology, Inc (Danvers, MA, USA).

## Results

### Cardiac miR-133a overexpression decreases mRNA levels of fibrosis markers in the diabetic mouse heart

**Figure 1:**
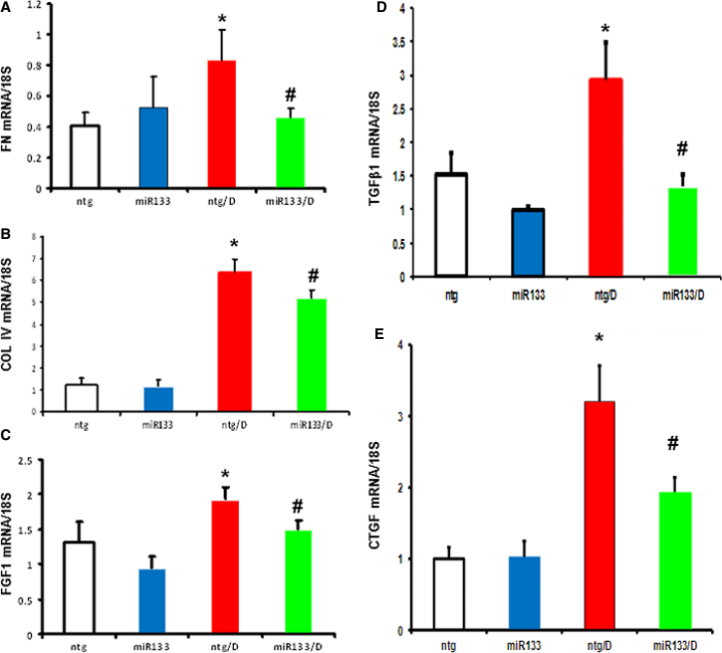
Effects of Cardiac miR-133 overexpression on fibrosis regulators during diabetes. Whole heart tissue from animals that had been diabetic for 2 months was analysed for mRNA levels of fibronectin (FN1; A), collagen (COL4A1; B), fibroblast growth factor1 (C), transforming growth factor-β1 (D) and connective tissue growth factor (E). Diabetes-induced up-regulation of these transcripts was prevented in the miR-133a animals. Expression data were normalized to that of the reference gene, 18S. *n* = 6–8/group; values are mean ± SEM. *Significantly different from ntg group, #significantly different from ntg/D group, *P* < 0.05.

We explored the influence of cardiac miR-133a overexpression on the expression of fibrosis markers. Four groups of animals were used: wild-type non-transgenic (ntg), non-transgenic with 2 months of streptozotocin (STZ)-induced diabetes (ntg/D), non-diabetic cardiac miR-133a TG (miR-133a) and cardiac miR-133a TG with STZ-induced diabetes (miR-133a/D). mRNA expression levels were determined for FN1 (Fig. 1A), COL4A1 (Fig. 1B), fibroblast growth factor1 (FGF1; Fig. 1C) and TGF-β1 (Fig. 1D) and connective tissue growth factor (CTGF; Fig. 1E), and levels were normalized to the reference 18S. Fibronectin, FGF1 and TGF-β1 showed an almost twofold increase in diabetic hearts, while COL showed a fivefold increase; the increases in FN1, FGF1 and TGF-β1 were attenuated in diabetic miR-133a overexpressed hearts. COL4A1mRNA was only moderately decreased, although this was significant. No changes in FN1 and COL4A1 mRNA levels were observed between the non-transgenic and miR-133a TG non-diabetic hearts, but TGF-β1 levels showed a decreasing trend.

### Cardiac miR-133 overexpression inhibits vasoactive mediators of fibrosis

**Figure 2:**
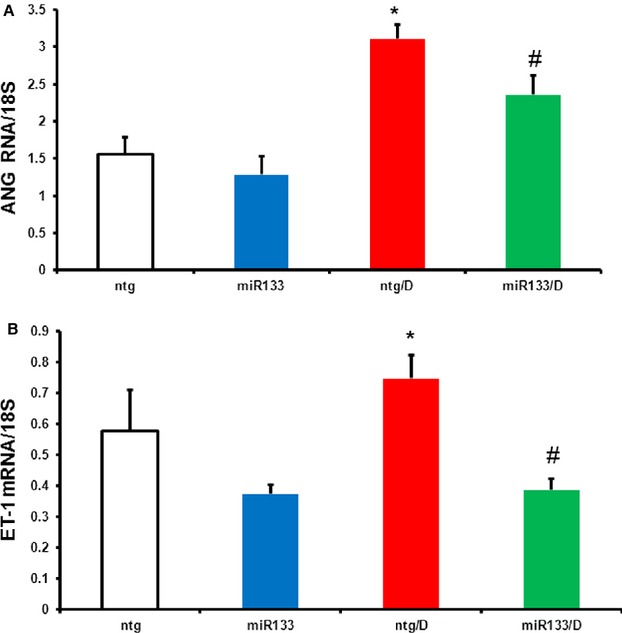
Regulation of angiotensinogen (AGT) and endothelin-1 (EDN1) mRNA expression during diabetes in the absence and presence of miR-133a overexpression. Hearts of the animals from the four groups (ntg, ntg/D, miR-133a and miR-133a/D) were isolated and mRNA expression for AGT (A) and EDN1 (B) for these groups was analysed by real-time PCR. Diabetes-induced up-regulation of these transcripts was prevented in the miR-133a animals. Expression data were normalized to that of the reference gene, 18S. *n* = 6–8/group; values are mean ± SEM. *Significantly different from ntg group, #significantly different from ntg/D group, *P* < 0.05.

Angiotensinogen (AGT) expression is required for production of Ang II and eventual activation of AT-receptor signalling. Angiotensin receptor over activation has been shown to play an important role in the pathogenesis of fibrotic events in various tissues [5, 12]. AGT is a precursor for Ang II, a major AT receptor ligand. We found that AGT mRNA was increased in the heart in diabetes (Fig. 2A) almost twofold and this effect was reversed by miR-133a overexpression (Fig. 2A), suggesting that miR-133a inhibits activation of the local renin–angiotensin system. Endothelin-1 is another prominent vasoactive substance involved in the regulation of fibrosis, through the involvement of its receptor, Endothelin-A [13]. Similar to AGT, we found that EDN1 mRNA levels were also increased in diabetes (Fig. 2B), an effect that was normalized in the miR-133a/D group (Fig. 2B).

### A reciprocal relationship of p300 and miR-133a was observed in the heart in diabetes

**Figure 3:**
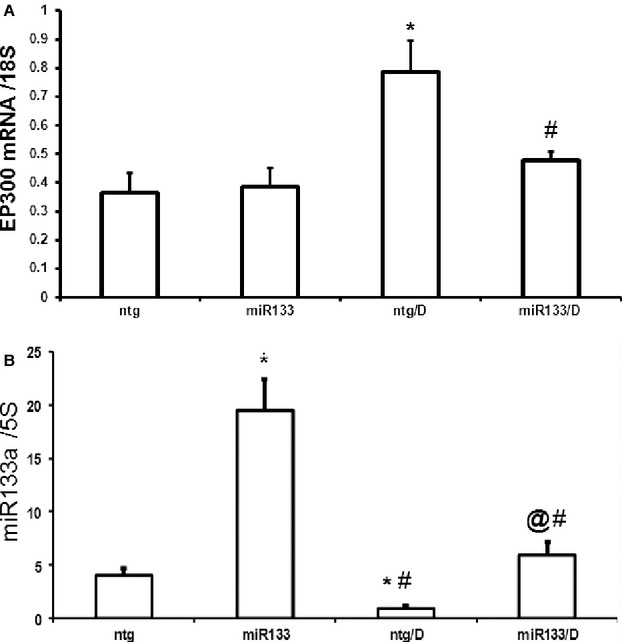
Influence of diabetes on miR-133a and EP300 mRNA levels in the heart. Diabetes (ntg/D) caused significant up-regulation of (A) EP300 mRNA expression in association with (B) reduced miR-133a expression in the hearts. Such alterations were prevented in the hearts of transgenic miR-133a animals with diabetes (miR-133a/D). 5SrRNA has been used for miR-133a and 18S was used as the loading control for EP300 mRNA, *n* = 6–8/group, values are mean ± SEM. *Significantly different from ntg group, &significantly different from miR-133a group, @different from all other groups, *P* < 0.05.

High glucose has been shown to regulate the insulin gene by hyperacetylation through the activity of the transcriptional co-activator, p300, in MIN6 cells [7]. Studies in our laboratory have also shown that high glucose increases p300 mRNA and protein levels in endothelial cell culture [8, 10]. In this study of STZ-induced diabetes, we observed that diabetes increased EP300 mRNA levels concurrent with a decrease in miR-133a in the heart (Fig. 3A and B) suggesting a possible negative regulation of miR-133a in diabetes. The increase in EP300 and decrease in miR-133a mRNA levels following diabetes were normalized to control levels in miR-133a TG diabetic mice (Fig. 3A and B).

### miR-133a overexpression in the heart mitigates diabetes-induced fibrosis

**Figure 4:**
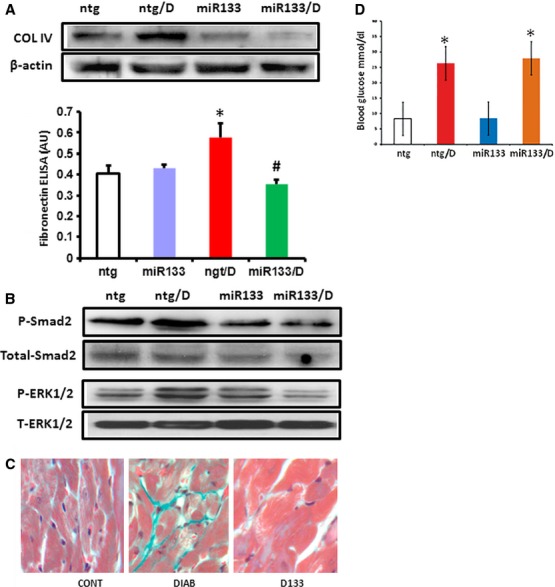
Protein expression changes and histological alterations following miR-133aa overexpression in diabetes. Protein levels of the major fibrosis markers were evaluated using (A) specific antibodies for COL4A1, and ELISA for FN1 and (B) antibodies against phospho-ERK1/2, SMAD and β-actin. Diabetes-induced specific protein up-regulation/and phosphorylation were prevented in the miR-133a animals. Results shown are representative of three to five different experimental sets; quantitative assessment of differential protein expression as observed in Western blot is shown in Figure S1. *n* = 6–8/group. The values are mean ± SEM. *Significantly different from ntg group, #significantly different from ntg/D group. (C) Histological sections of the heart showing diabetes; (D) induced focal fibrosis were prevented in the transgenic mice (miR-133a/D). Trichome stain was used for evaluation (green = fibrous tissue).

We then explored whether diabetes-induced stress was able to alter changes in levels of specific ECM proteins and signalling molecules of interest. Altered mRNA levels were reflected by altered abundances of ECM proteins in our experimental groups. We found that ntg/D group had increased protein expression levels for COL4A1 and FN1, which are markers of fibrosis (Fig. 4A). We have previously demonstrated that ERK activation plays a major role in increased ECM protein production in diabetes [8], which was also demonstrated in this study by phosphorylation levels of ERK (Fig. 4B). The role of TGF-β1 was established by drastically increased phosphorylation of SMAD-2, an immediate downstream target of TGF-β1 signalling, in diabetic (ntg/D) animals. The observed increases in COL4A1, FN1 and phosphorylation of both ERK1/2 and SMAD-2 were prevented when miR-133a was overexpressed in the heart in diabetes (Fig. 4B). Cardiac tissue sections were further examined Click here to enter text.for fibrotic lesions in the heart. Diabetic animals showed the presence of focal fibrosis using trichrome staining. Such changes were prevented in the miR-133a overexpressing animals with diabetes (Fig. 4C).

## Discussion

In this study, we have demonstrated that miR-133a overexpression can prevent diabetes-induced increased ECM protein expression and focal cardiac fibrosis. Cardiac fibrosis during diabetic cardiomyopathy is the end result of derangements in multiple signalling pathways [10, 13]. Increased expression of ECM proteins, FN1 and COL4A1, are hallmarks of cardiac fibrosis [10, 13]. In keeping with our previous studies [8, 14], we observed an increased accumulation of these proteins in the hearts of diabetic animals. Such accumulations were associated with an increased TGF-β1, AGT and EDN1 mRNAs.

We and others have previously shown the presence of focal fibrosis in diabetic cardiomyopathy and that there is increased expression of TGF-β1 signalling accompanied by enhanced COL4A1 and FN1 production [11, 15]. We [16]and others have also shown that EDN1 plays an important role in such process in diabetes through ERK1/2 activation [17] Furthermore, Kajstura *et al*. and Saulière *et al*. showed that AGT and its downstream mediators along with AT receptors play a significant role in this process [9, 18, 19].

In this study, we showed for the first time that miR-133a plays a specific role in the development of cardiac fibrosis in diabetes and indicate possible pathways involved in this process. In the heart, miR-1 and miR-133a are among the most cardiac-specific miRNAs that are abundantly expressed, and are known to regulate cardiac cell differentiation, survival and hypertrophic growth [20, 21].

We have shown down-regulation of miR-133a in the heart in diabetes [20]. In pressure overload hypertrophy, miR-133a overexpression can prevent cardiac fibrosis [20]. It was previously demonstrated that major targets of miR-133a include genes involved in development, cell cycle, cellular transport, metabolism and mitogenic signalling [21]. In keeping with such findings, this study also showed that overexpression of miR-133a prevents diabetes-induced increased ECM production leading to specific structural changes, *i.e*. focal myocardial fibrosis.

The exact mechanism of protection afforded by miR-133a is not clear. However, we found that miR-133a was able to decrease TGFβ1 mRNA expression, a known target of miR-133a (http;//www.targetscan.org). Transforming growth factor-β1 signalling-induced fibrosis has been shown to involve the ERK subfamily of stress kinases in kidney, liver and retinal epithelial cells, which we also observed in diabetic hearts [16]. We also observed an increase in acidic FGF1 mRNA in diabetes (another known target of miR-133aa) and an established activator of ERK1/2 phosphorylation [16]. Furthermore, recent work in fibroblasts has shown that AT-receptor stimulation also induces ERK phosphorylation [22]. Connective tissue growth factor is another important target of miR-133a, and we demonstrated a partial but significant reduction in its mRNA expression when miR-133a was overexpressed in the hearts of diabetic mice. Hence, a well co-ordinated axis exists among TGF-β1, EDN1, CTGF and AGT signalling, leading to activation of the histone acetyltransferase p300, *via* ERK signalling. EP300 acts as a transcriptional co-activator for multiple transcription factors [7, 13]. Our laboratory has previously shown that in endothelial cells and organs affected by chronic diabetic complications, including the heart, diabetes-induced increased ECM protein production is mediated through p300 activation [7]. Fibroblasts and skin biopsies from human cases showed an increased p300 expression and activity, following TGF-β incubation [23]. In our current study, we show that p300 mRNA levels increase with diabetes and this increase was correlated with an increase in mRNA levels of fibrosis inducers: EDN1, AGT, CTGF and TGF-β1, and ECM genes, *e.g*. COL4A1 and FN1.

**Figure 5:**
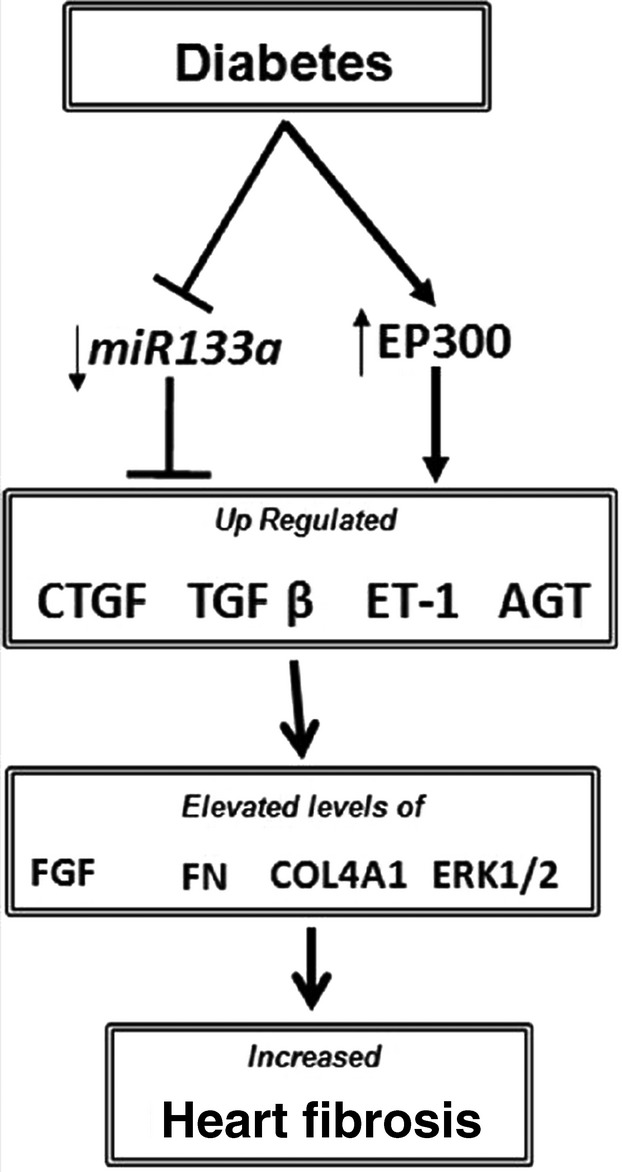
A diagrammatic outline of the findings showing potential mechanisms leading to cardiac fibrosis in diabetes. Hyperglycaemia is accompanied by an increased EP300 expression following by up-regulation of major regulators of fibrosis like transforming growth factor-β1, connective tissue growth factor, endothelin-1 and angiotensinogen. This in turn causes up-regulation of the mediators fibroblast growth factor1 and ERK1/2, which are turn on tissue fibrosis markers Collagen and Fibronectin. Diabetes is also accompanied by a decreased expressed of miR-133a. Interestingly, when animals were overexpressed with miR-133a in the heart, there was a significant attenuation of mediators and markers of fibrosis along with decreased fibrotic heart lesions.

Hyperglycaemia, through down-regulation of endogenous miR-133a expression, diminishes possible innate protection against fibrosis. The protective response we observed during miR-133a overexpression during diabetes involves down-regulation of TGF-β1 and decreased phosphorylation of SMAD2 and reduced ERK1/2 activation (Fig. 5 Summary Diagram). ERK1/2 activation in the context of reduced miR-133a in diabetes may further cause alteration of multiple fibrogenic factors and ECM protein production, leading to structural changes in the heart in diabetes. One notable observation is that miR-133a–mediated attenuation of fibrosis occurred even without reversal of hyperglycaemia (Fig. S2), which indicates that miR-133a could also become a therapeutic target along with or without insulin for treating diabetic patients. However, we do recognize the limitation of such studies as the demonstrated signalling mechanism may also represent an interplay of various cell types within the heart.

In summary, this study demonstrated a novel molecular mechanism by which miR-133a overexpression in the heart protects cardiac tissue from undergoing fibrosis during hyperglycaemia. We suggest that miR-133a could be a potential therapeutic target for combatting cardiac fibrosis.

## Supporting information

**Figure S1.**Quantification of the proteins in controls and miR-133a overexpressed mice with and without diabetes
