# Supplementary material for: Cardiac miR-133a overexpression prevents early cardiac fibrosis in diabetes
Source: J Cell Mol Med. 2014 Jan 16;18(3):415–21. doi: 10.1111/jcmm.12218 (PMC3955148; doi:10.1111/jcmm.12218)
Supplement: Supplementary file 2 [file jcmm0018-0415-sd2.docx]

Figure S1. Quantification of the proteins in controls and miR-133a overexpressed mice with and without diabetes
